# Predicting prediction: A systematic workflow to analyze factors affecting the classification performance in genomic biomarker discovery

**DOI:** 10.1371/journal.pone.0276607

**Published:** 2022-11-09

**Authors:** Michael Netzer, Christian Baumgartner, Daniel Baumgarten

**Affiliations:** 1 Institute of Electrical and Biomedical Engineering, UMIT - Private University For Health Sciences and Health Technology, Hall in Tirol, Austria; 2 Institute of Health Care Engineering with European Testing Center of Medical Devices, Graz University of Technology, Graz, Austria; King Abdulaziz University, SAUDI ARABIA

## Abstract

High throughput technologies in genomics enable the analysis of small alterations in gene expression levels. Patterns of such deviations are an important starting point for the discovery and verification of new biomarker candidates. Identifying such patterns is a challenging task that requires sophisticated machine learning approaches. Currently, there are a variety of classification models, and a common approach is to compare the performance and select the best one for a given classification problem. Since the association between the features of a data set and the performance of a particular classification method is still not fully understood, the main contribution of this work is to provide a new methodology for predicting the prediction results of different classifiers in the field of biomarker discovery. We propose here a three-steps computational workflow that includes an analysis of the data set characteristics, the calculation of the classification accuracy and, finally, the prediction of the resulting classification error. The experiments were carried out on synthetic and microarray datasets. Using this method, we showed that the predictability strongly depends on the discriminatory ability of the features, e.g., sets of genes, in two or multi-class datasets. If a dataset has a certain discriminatory ability, this method enables prediction of the classification performance before applying a learning model. Thus, our results contribute to a better understanding of the relationship between dataset characteristics and the corresponding performance of a machine learning method, and suggest the optimal classification method for a given dataset based on its discriminatory ability.

## Introduction

In genomics, high-throughput technologies such as DNA microarrays lead to huge amounts of data. Here, patterns of alterations in gene expressions are an important starting point for the discovery of new biomarkers [1]. In general, the underlying datasets are usually characterized by a large number of *features* (named variables or attributes) and *instances* (samples). Therefore, sophisticated machine learning approaches are required to search for new biomarker candidates in the data. Basically, *supervised learning approaches* can be divided into *regression* (dependent variable is continuous) and *classification* (dependent variable is categoric) methods. Since biomedical datasets are often characterized by categorical class variables, e.g., for predicting cases versus controls, in this work we focus mainly on classification methods, of which there is a wide variety of models. The aim of classification is to predict a class (e.g., diseased and healthy controls) of a new unclassified instance based on a set of features (e.g., gene expression levels). The model is learned from a training dataset $TR={\left\{x_{i},y_{i}\right\}}_{i}^{n_{TR}}$, where *n*_*TR*_ is the number of training instances (samples), *x*_*i*_ is a *d*−dimensional feature vector and *y*_*i*_ is the corresponding class label.

The underlying theoretical principles of the learning approaches were introduced by Valiant [2] in 1984 and are known as p*robably approximately correct* (PAC) learning. The theory was later extended to include a simple combinatorial parameter called the Vapnik Chervonenkis dimension [3].

In practice, a common approach is to examine the classification error of different classification models and select the method with the highest performance. For example, Hsieh et al. [4] compared four learning models for predicting the mortality of patients with unplanned extubation in intensive care units or Kim et al. [5] for the prediction of glaucoma. In the area of text classification, Onan et al. [6] compared base learning methods with ensemble methods for extracting important keywords using the ACM document collection or more recently in a biomedical domain using public biomedical text categorization datasets [7]. However, the relationship between data set features and the performance of classification is not yet fully understood.

In contrast to our previous works on performance evaluations of learning models (e.g. [8, 9]), in this research we aim to better understand and quantitatively evaluate the relationship between dataset characteristics in terms of discriminatory ability and associated classification performance of the applied supervised learning methods. A novel multi-step workflow is proposed to analyze and estimate the predictability of different classification models based on the dataset characteristics using synthetic and experimental microarray data before applying any learning model. To the best of our knowledge, our proposed workflow is the first in the field of biomarker discovery to predict the prediction performance of common classifiers before their application.

## Computational workflow

We present here a three-step workflow to estimate and evaluate the predictability of a classifier’s classification performance. In a first step, we import the raw datasets. Next, we infer *p* features that characterize the given number of datasets *n* of a given domain. These *characterizing features* can be divided into three groups:

1. Features describing the univariate discriminatory ability
2. Features describing the correlation structure of the dataset
3. Features describing the presence of possible sub cluster structures

**Fig 1:**
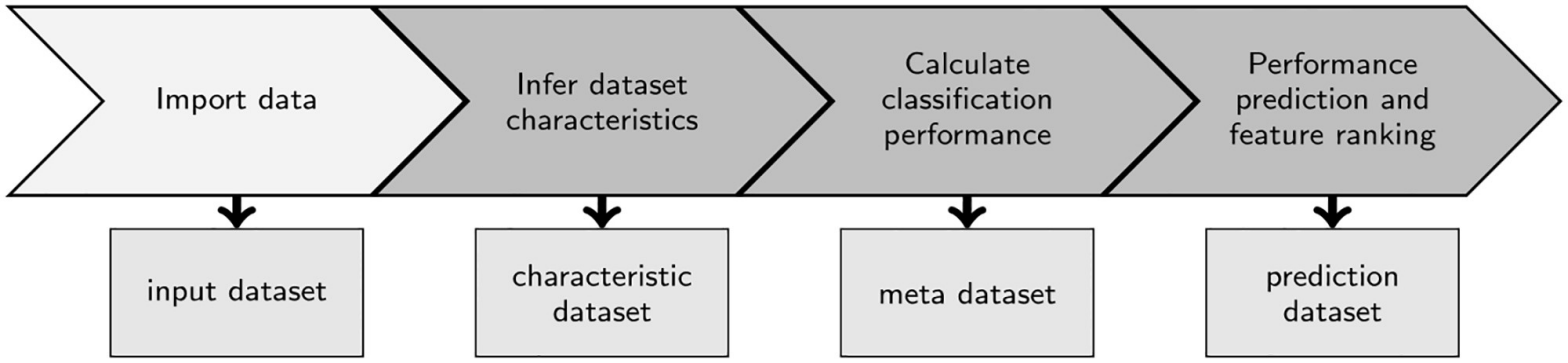
Computational workflow. Data import and the proposed three-step computational workflow. The boxes below the workflow show the actual output of each step.

The resulting dataset is called a *characteristic dataset*. Next, we construct a so-called *meta dataset* that combines the dataset features $X\in R^{p}$ with the classification performance values Y∈R of the initial input dataset in terms of the classification error. Now this is a classic machine learning problem, where we search a function *f*(*X*) that predicts *Y* (dependent variable, here a vector of classification errors) based on the dataset features *X* (independent variables). A regression analysis is carried out to find a function for predicting the classification errors *Y*. In a final step, the prediction performance of the chosen regression method is determined and the features are ranked according to their impact on the prediction of the resulting classification error. Fig 1 shows the developed workflow. The individual steps are described in more detail in the following sections.

### Step I: Infer dataset characteristics

Basic dataset characteristics include the number of samples and features, the type of the independent variables (e.g., categorical or numeric) and the type of the dependent variable to be predicted. It is known that feature characteristics such as effect size and discriminator ratio have a great impact on the performance of supervised machine learning methods [8] and that correlations between the features significantly affect the classification results [10]. Therefore, this information is also important for predicting the prediction error.

#### Univariate discriminatory ability

We applied filter-based feature selection methods to quantify the univariate discriminatory ability of the individual features in a dataset. These methods estimate the usefulness of features using evaluation metrics [11]. Specifically, Information Gain [12], ReliefF [13] and the t-value of a Student’s t-Test were selected as filter methods. Table 1 summarizes the selected parameters, including location, dispersion, shape, p-value based and other descriptive parameters.

**Table 1:** Description of the statistical parameters of the filter scores.

Parameter	Description
Location parameters	Mean, trimmed mean, median
Dispersion parameters	Standard deviation, median absolute deviation
Shape parameters	Skew, kurtosis
P-value based statistics	Number of features with a p-value/adjusted p-value lower than 0.001, 0.01, 0.05, 0.1. In addition, the total number of features below the thresholds was calculated. The adjusted p-values were calculated using the Benjamini & Hochberg procedure.
Further parameters	Number of valid cases, minimum, maximum, standard error, percentiles

#### Correlation structure

A network-based approach was introduced to capture the correlation structure of the dataset. Let *G* = (*V*, *E*) be an undirected graph, where *V* is the set of vertices in *G*, and *E* is the set of unordered pairs of elements of *V* [14]. The vertices of correlation networks here represent features. The edges of the network represent correlations between these features. After inferring the network, common topological global graph descriptors are calculated. Table 2 shows the topological descriptors used. For a more detailed and comprehensive description of these descriptors see [15].

**Table 2:** Description of global graph descriptors (see also [15]).

Parameter	Description
Graph density	Ratio of the number of edges and all possible edges
Reciprocity	Proportion of mutual connections
Transitivity	Ratio of connected triples in the graph
Diameter	Maximal distance in a graph
Mean distance	Mean path length in a graph

#### Sub cluster quantification

It is known that the presence of sub clusters in the dataset can affect classification performance. For example, if the class variable is binary (e.g., diseased and healthy) additional sub clusters can exist in each of these groups and can affect the classification performance. To identify these sub clusters, the following processing steps were performed: i) application of a partition based clustering method such as *k*-Means to obtain clusters for varying *k*, and ii) calculation of cluster indices. These indices allow to estimate the number of existing clusters (e.g., where C-index exhibits a minimum value).

### Step II: Classification

We applied four classification methods commonly used in biomedical data analysis.

*K-Nearest Neighbors (KNN)* [16] is a simple instance-based learning method, where the class prediction of a newly unclassified instance is determined by considering the majority of *k*-nearest instances. Euclidean distance is typically used as a distance metric when continuous features are present.

*Linear Discriminant Analysis (LDA)* [17] seeks a projection hyperplane that minimizes the interclass variance and maximizes the distance between means of classes [18]. An observation *x*_*i*_ is assigned to a class *y* by projecting data onto a single dimension defined by [19]

$$\begin{array}{c} \Sigma^{-1}\left(\mu_{1}-\mu_{2}\right), \end{array}$$ (1)

where Σ is the covariance matrix and *μ*_*i*_ are mean values of the corresponding classes.

*Support Vector Machine (SVM)* [20] establishes a maximum-margin separating hyperplane with normal vector *w* between classes by solving expression (2) [21]

$$\begin{array}{c} \underset{w}{\text{min}}({\left||w|\right|}^{2}+C\sum_{i=1}^{n_{TR}}\xi_{i}),\\ \forall i\in 1,2,\ldots ,n_{TR}:y_{i}w^{T}x_{i}\geq 1-\xi_{i},\\ \xi_{i}\geq 0, \end{array}$$ (2)

where *C* is a trade-off parameter used to penalize misclassifications, and *ξ*_*i*_ are slack variables that control the degree of misclassification. Without extension, SVM are designed for binary classification problems where *y*_*i*_ is a binary label (e.g, + 1 and −1 or + 1 and 0 respectively). Projections into higher dimensional spaces allowing the data to be linearly separated [22].

*Random Forests (RF)* is an ensemble method that combines single decision trees from independently subsamples of a dataset and are generally robust to noise [23]. RF also captures complex dependency patterns between dependent and independent variables [24]. This method is characterized by high performance and was recently used successfully to predict delirium in gerontopsychiatric patients [25].

To estimate the generalization performance of a learning model, the data set can be split into a training (*TR*) and a test (*TE*) dataset (e.g., 60% of the samples are used for training and the remaining samples for testing). However, to reduce the bias of samples selected for testing, this process must be repeated. A recommended validation strategy is 10-fold cross-validation [26]. Here, the dataset is divided into 10 partitions, where 9 partitions are used for training and the remaining part for testing, this 10 times. The resulting mean performance value and standard deviation can be used to more realistically estimate the predictive ability of the classifier. In our approach, we also chose a 10-fold cross-validation strategy.

### Step III: Performance prediction and variable importance

Three regression models were used to predict the classification error: linear regression (LR), random forest (RF) and Bayesian generalized linear models (BG) [27]. The corresponding tuning parameter *mtry* represents the number of features randomly sampled at each node to be considered for splitting [28] and was identified using hyperparameter tuning. The final performance of all regression models with respect to *R*^2^ was calculated on an independent test set with 25% of hold-out samples. Feature ranking was calculated within the cross-validation loop using an embedded feature selection approach. In particular, variable importance was determined using the absolute value of the t-statistic for the linear regression model and the mean decrease in node impurity for the random forest model (function varImp in *caret*-package, see also [29]). In an additional analysis, we compared the similarity of rankings between our simulations with Spearman’s correlation.

### Workflow implementation

All methods were implemented with the programming language *R* [30]. We used the function classificationError of the R-package *optBiomarker* [31] which considers the four base classifiers RF, SVM, LDA and KNN. Performance prediction with LR and RF regression was conducted using the *caret* [29] package.

The scores resulting from the filter approaches were summarized with the statistical function describe of the R-package *psych* [32] in order to estimate the discriminatory ability of a dataset. Topological global graph descriptors were computed using the R-package *igraph* [15]. Cluster indices were determined with the R-package *clusterCrit* [33]. A summary of the indices can be found in the corresponding package descriptions.

## Experiments on synthetic and biomedical data

### Synthetic data

The R-package *optBiomarker* [31] was used to generate the synthetic datasets. In particular, we specified the number of cases, features, artificial “biomarkers” (discriminators), effect sizes and correlation structure using the method described in [34]. The distribution of non-discriminating features was defined by $N(0,\;\sigma^{2})$, where *σ*^2^ represents the combination of technical standard deviation (*sdW*) and biological standard deviation (*sdB*). Accordingly, discriminators were simulated by adding *zδ* to the values of the control group. The parameter *δ* results from a truncated normal distribution and *z* is randomly selected between -1 and 1 to simulate up- or down-regulation, respectively [31, 34]. We extended the configuration method by enabling the combination of different configuration parts with varying parameter settings (see Table 3).

**Table 3:** Configuration reference file consisting of three parts (P1, P2, P3) with varying minimum (2, 2 and 1) value of fold changes (*foldMin*).

	Parameter	P1	P2	P3
1	num.datasets	300.00	300.00	300.00
2	*n*_*TR*_	100.00	100.00	100.00
3	nGr1	50.00	50.00	50.00
4	nBiom	50.00	20.00	20.00
5	nRep	3.00	3.00	3.00
6	sdW	3.00	3.00	1.00
7	sdB	3.00	3.00	1.00
8	bsMin	3.00	3.00	3.00
9	sigma	0.10	0.10	0.10
10	diffExpr	1.00	1.00	0.00
11	foldMin	2.00	2.00	1.00
12	orderBiom	1.00	1.00	1.00

The overall number of datasets was 900. Each dataset includes 100 samples (*nTrain*) divided into 2 equally sized groups with 50 samples (here *nGr1* = *nGr2*). The overall number of discriminators (*nBiom*) is 90. The number of technical replications (*nRep*), minimum block size (*bsMin*), technical (*sdW*) and biological (*sdB*) variation was set to 3. The standard deviation of the normal distribution (*sigma*) was set to 0.1 (default value). The systematic difference between groups (*diffExpr*) was enabled for P1 and P2. All columns were sorted in order of differential expression (*orderBiom*). Table 4 summarizes the change of simulation parameters compared to the reference simulation *S*1.

**Table 4:** Summary of the simulations used in this study. The descriptions of simulation *S*2 to *S*9 outline the changes compared to the reference simulation *S*1.

Simulation	Description
S1	Reference simulation
S2	Half number of samples in nTrain
S3	Half size of num.datasets
S4	Smaller fold changes (2,1.5,1 instead 2,2,1)
S5	Smaller fold changes (1.5,1,0.5 instead 2,2,1)
S6	Smaller number of discriminators (25,10,10 vs. 50,20,20)
S7	Higher and fixed correlation (rhoMax u. rhoMin)
S8	Increased biological variation (4,4,2 instead 3,3,1)
S9	Higher biological variation and smaller fold min

### Microarray data

DNA microarrays enable expression levels of thousands of genes to be measured simultaneously to identify patterns specific to serious diseases such as cancer. In our study, we used cancer microarray datasets from the Curated Microarray Database (CuMiDa) that underwent background correction, normalization and quality analysis [35]. The inclusion criteria for the studies considered were i) no chemotherapics and gene therapies including interfering molecules such as miRNA, siRNA, ii) only human studies, iii) no knockdown cultures or induced mutations, iv) at least six samples per condition, v) reasonable protocols, vi) no used xenograft technique and vii) availability of raw data [35]. Microarray data was obtained from platforms of Illumina, Agilent, and Affymetrix resulting in 78 selected data sets. Finally, from these 78 datasets we selected *n*_*d*_ = 37 datasets by using only binary classes with at least 10 samples in the control group. The cancer types available were breast, colorectal, gastric, leukemia, liver, lung, pancreatic, prostate, renal and throat cancer. The corresponding number of datasets considered for each cancer type can be found in Table 5.

**Table 5:** Number of datasets (*n*_*d*_) for each cancer type.

Cancer type	*n*_*d*_
Breast	3
Colorectal	5
Gastric	1
Leukemia	4
Liver	7
Lung	6
Pancreatic	1
Prostate	5
Rrenal	2
Throat	3

We transformed expression values using the binary logarithm. This is a common procedure to symmetrize expression ratios by symmetric treatment of numbers and their reciprocals [36]. We then selected a proportion of 10% highly variable genes to reduce the computational efforts for further analyses (see also [37] for more information). In general, a balanced class distribution is important to obtain a robust classification scheme [38]. Consequently, the datasets were balanced using the *ROSE* package [39].

## Results

### Performance prediction

**Fig 2:**
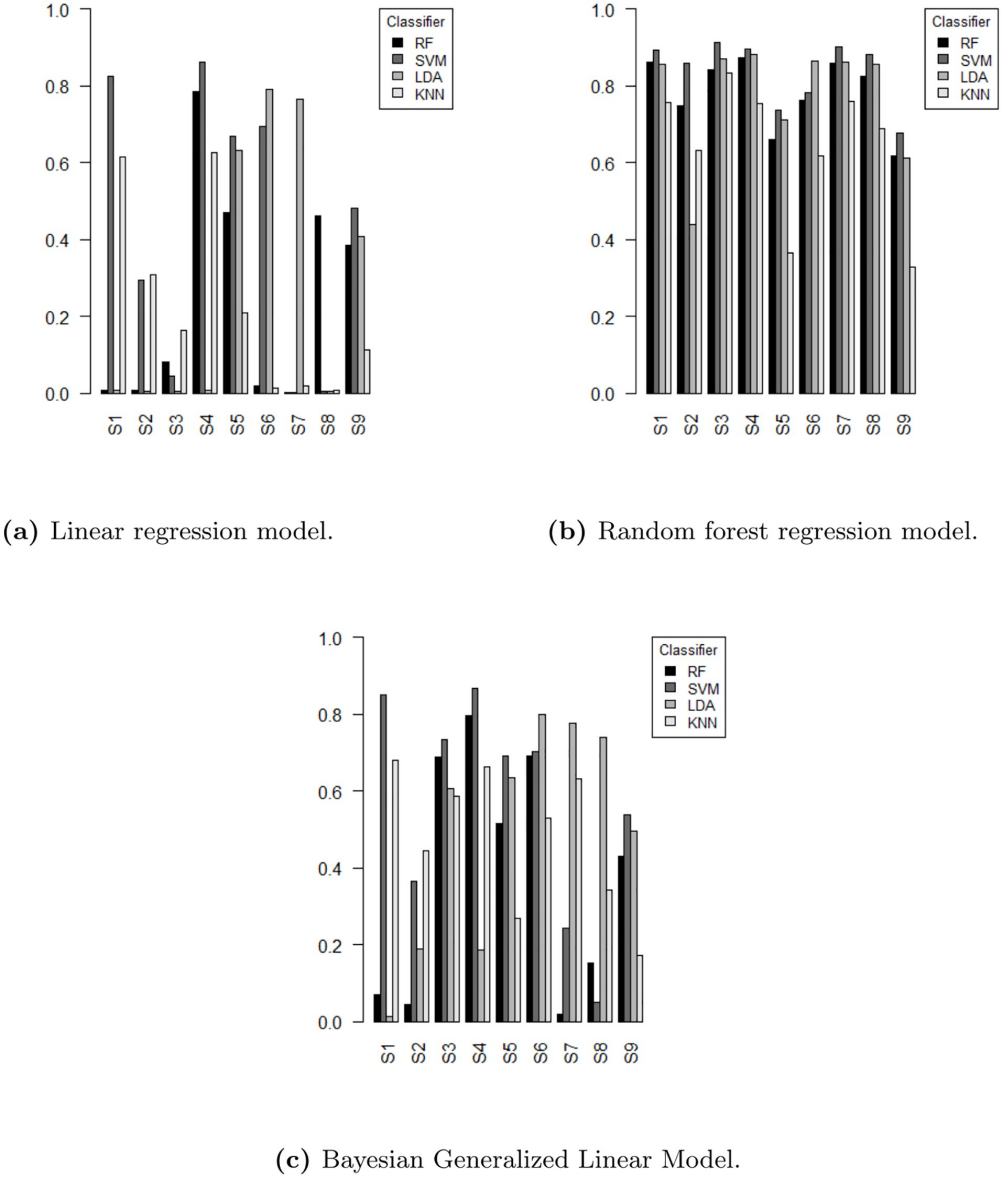
Prediction performance of the synthetic dataset. *R*^2^ to predict the classification errors of all considered simulations and classifiers using a linear regression model (left) and random forest regression model (right) and Bayesian Generalized Linear Model (bottom). *R*^2^ values were calculated using an evaluation set.

Fig 2 shows the prediction performance in terms of *R*^2^ for all simulations for the considered classifiers RF, SVM, LDA and KNN for the **synthetic data**. We used linear regression, random forest and Bayesian Generalized Linear Model as regression models to predict the classification error. Final *R*^2^ values were calculated using an evaluation set of 25% hold-out samples. For example, the first gray bar of the left figure indicates that the linear regression model is able to predict the accuracy of the SVM classifier with *R*^2^ larger than 0.8. Predicting the KNN classification error was most difficult, resulting in small *R*^2^ values. In particular, the *R*^2^ values for *S*9 were low (mean *R*^2^ < 0.6 for all regression models). Note that simulation *S*9 is characterized by higher biological variation and smaller fold changes. When comparing the dispersion of classifiers, *R*^2^ values among the simulations KNN and LDA were characterized by higher uncertainties compared to RF and SVM. Comparing the dispersion of the regression methods, the *R*^2^ values of the linear regression model showed higher variances.

In summary, the graph shows considerably higher performance values in terms of *R*^2^ using the random forest regression model. For example, the *R*^2^ values of the four base classifiers for simulation *S*1 ranged from 0.01 to 0.83 for the linear, from 0.76 to 0.89 for the random forest regression model and between 0.01 and 0.85 for the Bayesian model, respectively. Similar results were also obtained for the remaining simulations *S*2 to *S*9. Table 6 shows the results of the random forest regression model for predicting the classification error on the training set for reference simulation *S*1 using hyperparameter tuning. The tuning parameter *mtry* only marginally affected the performance of the random forest model (i.e., maximal absolute change of *R*^2^ ≤ 0.01). The best performance was observed predicting the classification error of SVM (*R*^2^ ≥ 0.9). Table 7 shows the *R*^2^ values for simulation *S*1 using the tuned model on the evaluation set.

**Table 6:** Root mean squared error (RMSE), *R*^2^ and corresponding standard deviation (*R*^2^SD) of the regression analysis (random forest model) for prediction classification errors of RF, SVM, LDA and KNN on the synthetic dataset. Metrics were calculated on the training dataset for a different number of predictors (mtry) using 10-fold cross-validation.

Classifier	mtry	RMSE	*R* ^2^	*R*^2^ SD
**RF**	2	0.04	0.86	0.01
	88	0.04	0.87	0.02
	175	0.04	0.87	0.02
	261	0.04	0.87	0.02
	348	0.04	0.87	0.02
**SVM**	2	0.05	0.9	0.02
	88	0.04	0.91	0.01
	175	0.04	0.91	0.01
	261	0.04	0.91	0.01
	348	0.04	0.91	0.01
**LDA**	2	0.04	0.86	0.02
	88	0.04	0.87	0.01
	175	0.04	0.87	0.01
	261	0.04	0.87	0.01
	348	0.04	0.87	0.01
**KNN**	2	0.05	0.74	0.02
	88	0.05	0.75	0.02
	175	0.05	0.74	0.02
	261	0.05	0.74	0.02
	348	0.05	0.74	0.02

**Table 7:** Performance values in terms of *R*^2^ for predicting the classification error of RF, SVM, LDA and KNN using linear regression, random forest regression and Bayesian Generalized Linear Model models. *R*^2^ values were calculated using the test set with hold-out samples.

	*R* ^2^		
Model	Linear Regression	Random Forest	Bayesian GLM
RF	0.01	0.86	0.07
SVM	0.83	0.89	0.85
LDA	0.01	0.86	0.01
KNN	0.61	0.76	0.68

**Fig 3:**
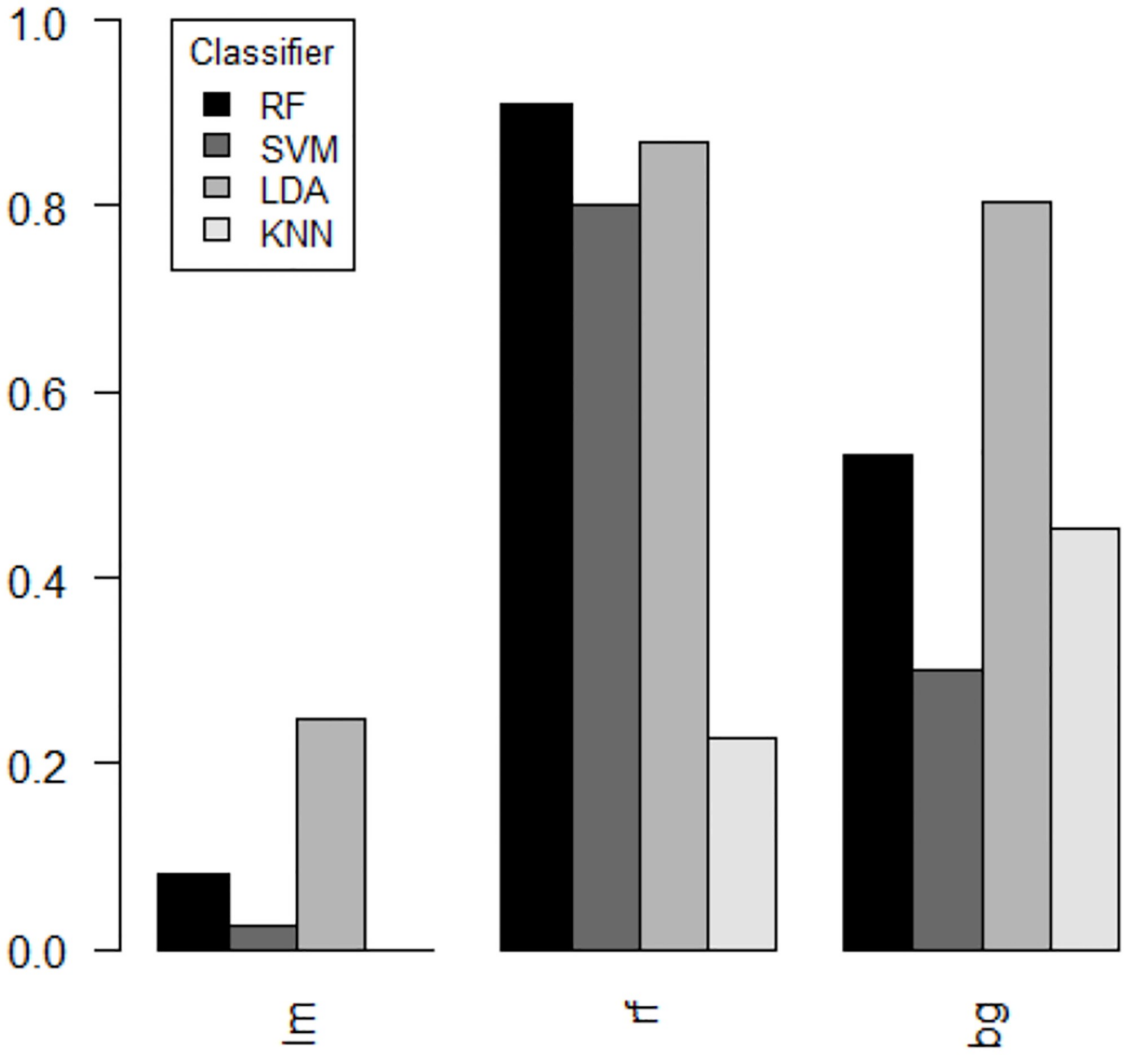
Prediction performance of the microarray dataset. Performance values in terms of *R*^2^ using a linear regression model (lm), random forest regression (RF) and a Bayesian Generalized Linear Model (bg) regression model using the microarray dataset. *R*^2^ values were calculated using the test set with hold-out samples.

A similar result was obtained for the **microarray dataset**. Overall, the *R*^2^ values were smaller compared to the synthetic dataset (*R*^2^ from 0.00 to 0.25 for the linear, from 0.30 to 0.81 for the Bayesian and between 0.23 and 0.91 for the random forest regression model, see Fig 3 and S1 Table). As observed for the synthetic dataset, predicting the KNN classification error using the linear regression model was most difficult. The best prediction performance in terms of *R*^2^ was obtained for predicting the classification error of RF using a random forest regression model.

### Feature ranking

**Fig 4:**
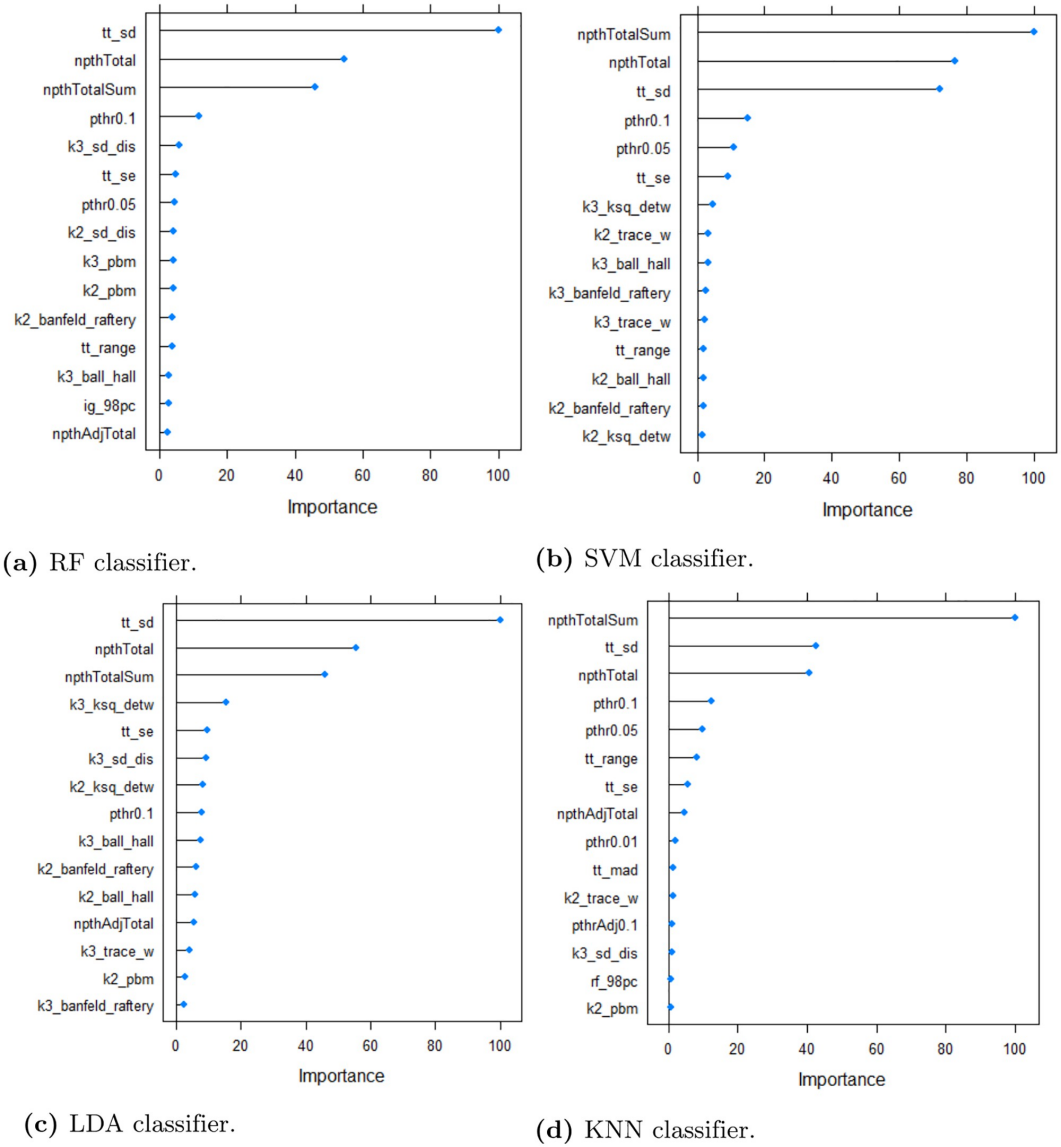
Feature ranking of the synthetic dataset. The top 15 ranked features with the highest variable importance using RF regression model using the synthetic dataset. The features include measures based on t-statistics such as the standard error (*tt*_*se*) and deviation (*tt*_*sd*) of t-values, *k*^*th*^-percentiles of Information Gain (e.g., *ig*_98*pc*) and ReliefF (e.g., *rf*_98*pc*) and cluster indices based on the number of *k* clusters (e.g., *k*3_*ball*_*hall* representing the Ball-Hall index for *k* = 3 clusters).

**Fig 5:**
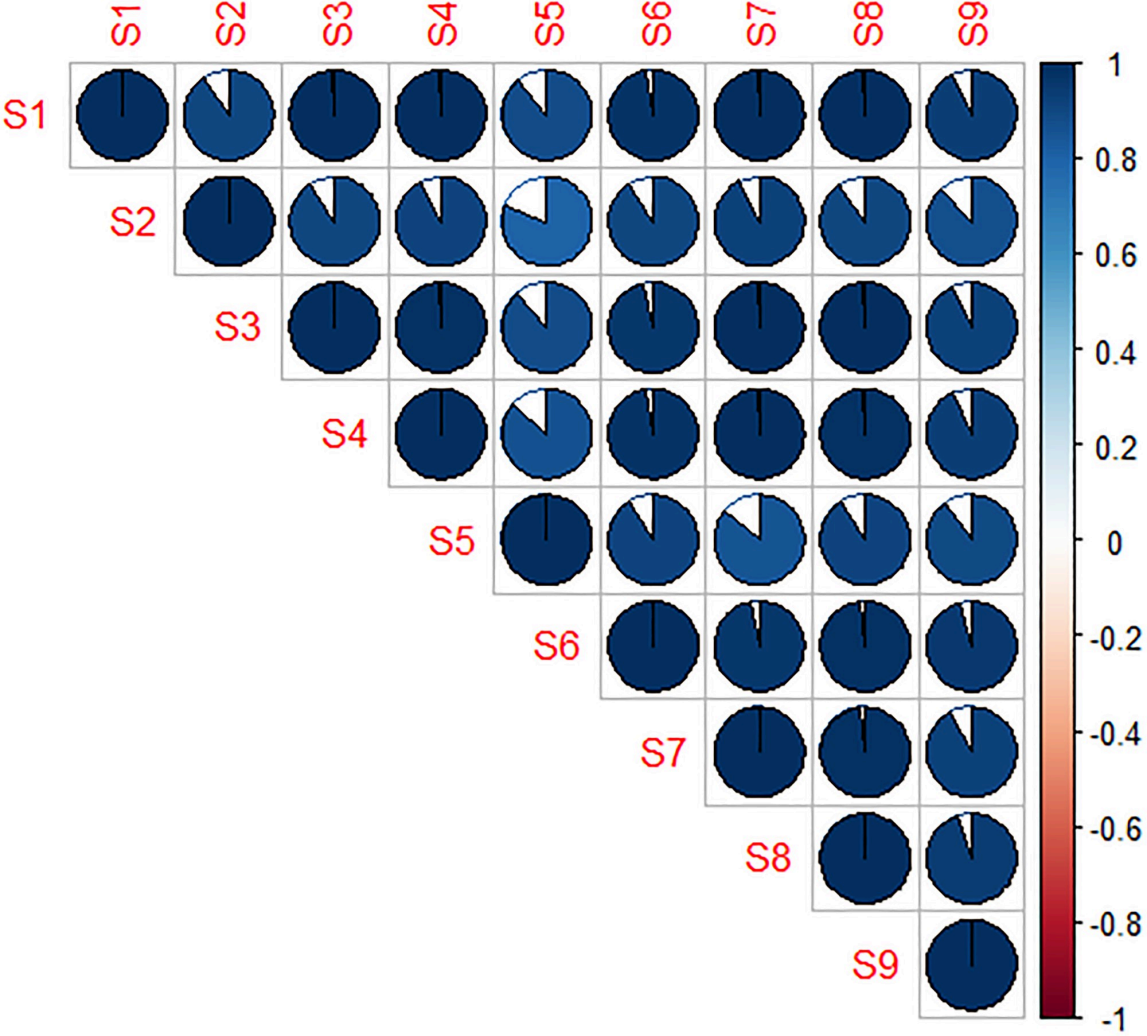
Correlation plot. Plot visualizing the correlation between feature scores of our simulations (linear regression model).

The top 15 ranked dataset features of simulation *S*1 of the **synthetic dataset** are shown in Fig 4. Variable importance represented the normalized differences in mean squared error calculated using the out-of-bag data for each tree and after permuting the corresponding variable [29]. Feature *npthTotal* was among the top three ranked features that predicts the classification errors using RF, SVM, LDA and KNN. In particular, this dataset feature represents the number of features with a p-value below *α*. Parameter *tt*_*sd* was also among the top three ranked features. This measure represents the variance of the t-values of the Student t-test. We analyzed the correlation between rankings of the nine simulations. Fig 5 visualizes the correlation between the simulations indicating a generally high similarity in feature rankings. The degree of filling of each circle indicates the degree of correlation between the respective simulations. In addition, the color represents the sign of the correlation value (i.e., positive or negative scores). The plot shows positive correlation scores greater than 0.75 among the nine simulations. According to this presentation, the rankings of simulation *S*2, *S*5 and *S*9 in particular deviate from other simulations. These experiments are characterized by smaller sample size (*S*2), smaller fold changes of biomarkers (*S*5) and higher biological variation (*S*9).

**Fig 6:**
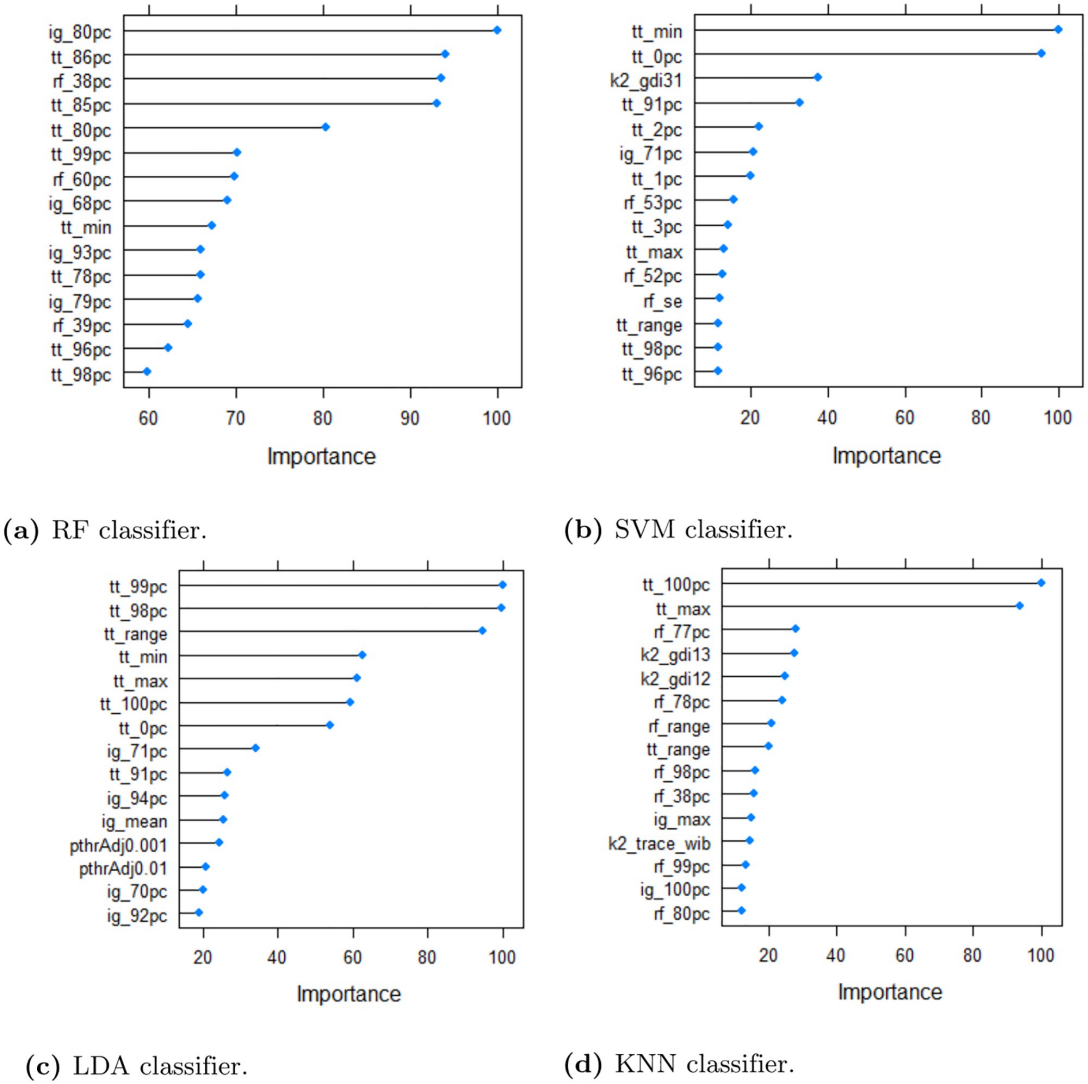
Feature ranking of the microarray dataset. The top 15 ranked features with the highest variable importance using RF regression model using the microarray dataset. The majority of features include measures based on t-statistics such as minimum (*tt*_*min*), maximum (*tt*_*max*), mean (*tt*_*mean*), median (*tt*_*median*), range (*tt*_*range*) and further *k*^*th*^-percentiles. Again, the prefixes *ig* and *rf* denote the filter-based methods Information Gain and ReliefF, respectively. The prefix *pthrAdjT* corresponds to the number of features with an adjusted p-value <*T*. For example, *pthrAdj*0.001 refers to the number of features with an adjusted p-value <0.001. The prefix *kc* corresponds to cluster indices for a specified number of clusters *c*. For instance, *k*2_*gdi*31 refers to Generalized Dunn Index with a between-group distance of 3 and a within-group distance of 1 using a k-Means clustering with *k* = 2 clusters. For a detailed overview of cluster indices see R-package *clusterCrit* [33].

Fig 6 shows the 15 top ranked features of the **microarray dataset**. Most of the features included distribution measures of the t-values such as different percentiles (e.g., *tt*_92 represents the 92 percentile of t-values). Interestingly, in contrast to the synthetic data the number of cluster indices was significantly smaller. When comparing the absolute scores of the variable importances of the synthetic and microarray dataset, the differences between the scores of the second one were smoother and more regular. Again, the importance scores of the synthetic dataset of the top ranked features were predominant for determining the classification error. The remaining features contributed only to a lesser extent to the performance of the regression model.

### Use case

**Fig 7:**
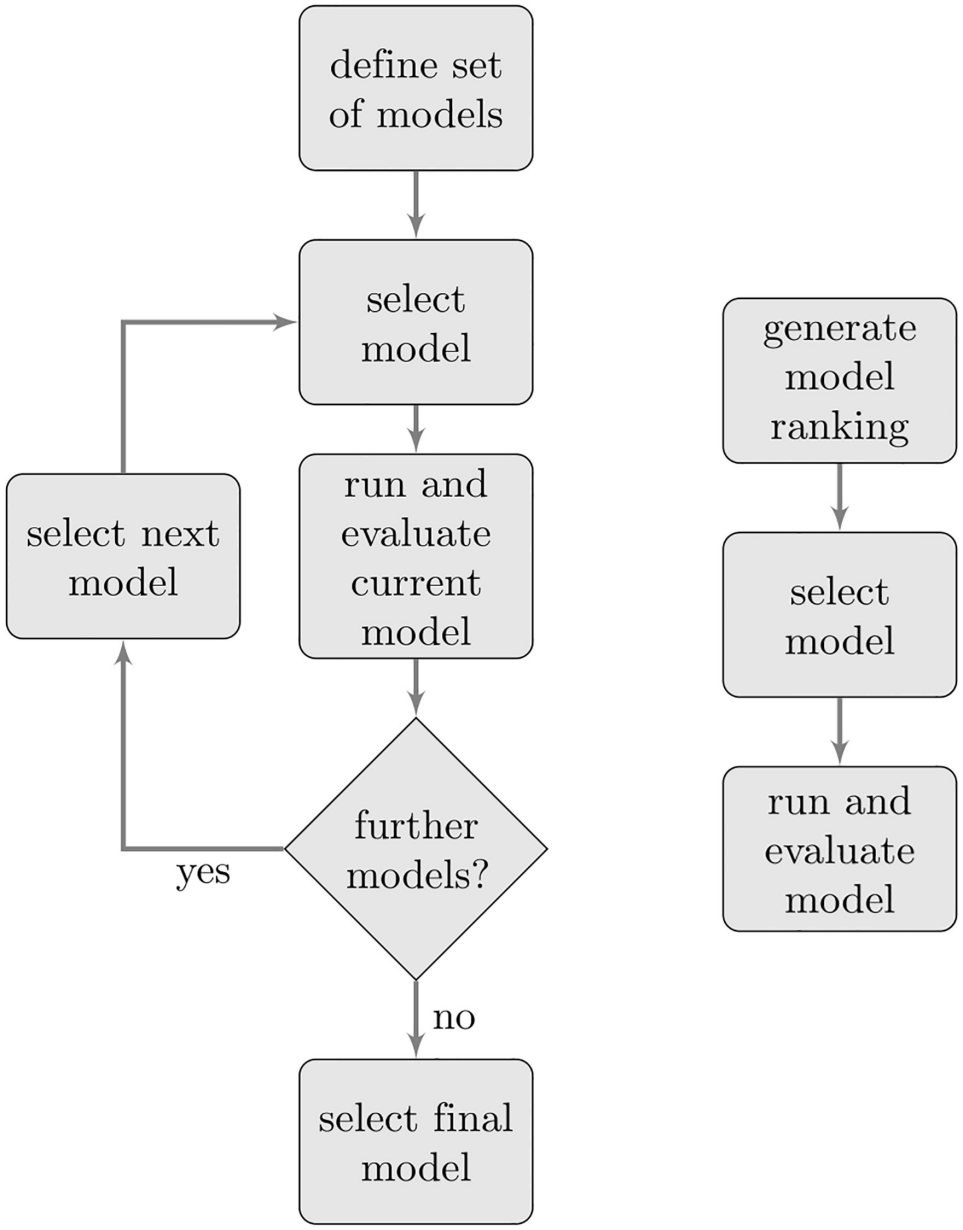
Workflow comparison. Comparison of the classical machine learning workflow (left) and our proposed approach (right).

**Fig 8:**
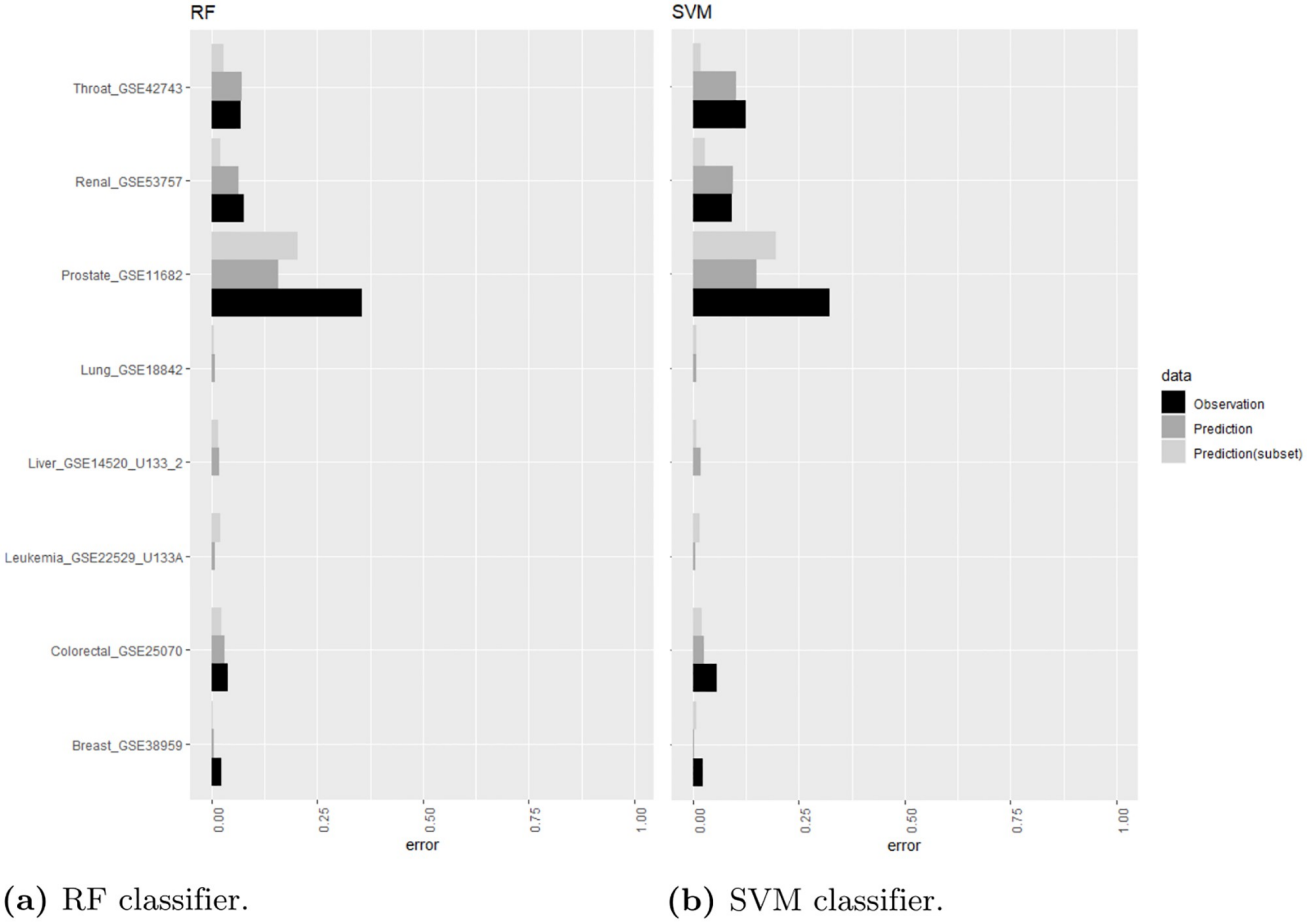
Comparison of performance prediction. Comparison of the true/observed (black bars) and predicted (light and dark gray bars) classification error using our approach. The left plot corresponds to the classification errors of the RF model and the right plot to classification errors of the SVM. The predicted errors using all characteristic features are visualized in dark gray and using only a subset of t-test based features in light gray. The prediction was built on a RF regression model.

Predicting serious diseases such as cancer based on DNA microarray data is a typical use case for machine learning in genomics. After preprocessing, a data scientist usually applies different machine learning classification models and compares the performance metrics (e.g., accuracy). In contrast, our proposed approach allows predicting the performance of different machine learning classification methods before any of them are applied. The considered classifiers can be ranked according to their predicted performance. The final classification method can then be the selected, taking into account further aspects such as the interpretability of the model. Fig 7 compares the typical machine learning workflow with our proposed method. Note that our approach can be also embedded in an automated machine learning process that includes hyperparameter preparation and tuning. For our application example, we used eight microarray datasets each representing one cancer type as independent test set (22% of all instances). Table 8 depicts the randomly selected test instances and the corresponding ID. The remaining datasets were used to train the model (78% of all instances). Fig 8 shows the observed and predicted classification errors using a RF and SVM classifier. Performance predictions were made with a RF regression model using i) all characteristic features and, ii) only a subset of t-test based features. The corresponding resulting root mean squared errors (RMSE) for predicting results of a RF classifier were 0.07 (all features) and 0.06 (t-test based features). Similar RMSE values were also obtained for predicting classification error of a SVM (RMSE = 0.06 for all features, RMSE = 0.06 using only t-test based features).

**Table 8:** Considered microarray test datasets for different cancer types and corresponding ID.

Cancer type	ID
Breast	GSE38959
Colorectal	GSE25070
Leukemia	GSE22529_U133A
Liver	GSE14520_U133_2
Lung	GSE18842
Prostate	GSE11682
Renal	GSE53757
Throat	GSE42743

It is important to note that the required computational time for inferring the dataset characteristics and predicting the error is much lower when only t-test based features are used. For instance, for the breast cancer dataset *Breast*_*GSE*38959 the method classificationError of the R-package *optBiomarker* took 1183.87 seconds to process the dataset (43 samples, 1716 features) using RF, SVM, LDA, and KNN on an Intel(R) Core(TM) i5–6200U CPU with 16 GB RAM. In contrast, computing all filter-based measures based on Student t-test took only 1.35 seconds. Note that t-test based features make up the majority of the 15 top ranked features (see Fig 6 and Table 9) for predicting the classification error of the considered classifiers. The highest ratio of t-test based features was observed for the LDA classifier, which has similar normality assumptions.

**Table 9:** Number of the t-test based features among the top 10 best ranked features for predicting the classification error of RF, SVM, LDA and KNN (microarray dataset), see also Fig 6.

Classifier	Number of t-test based features
RF	5 of 10
SVM	7 of 10
LDA	8 of 10
KNN	3 of 10

## Discussion and conclusion

In this work, we introduced a three step computational workflow to estimate and evaluate the relationship between dataset properties and the performance of supervised learning approaches. We have applied this approach to artificially generated data sets representing the characteristics of biomolecular data typically generated in genomic applications as well as real-world data from microarray experiments. In a first step, features were extracted that quantify basic dataset characteristics, including univariate discriminatory ability, correlation and sub cluster structure. Next, a *meta dataset* consisting of features describing the dataset characteristics and the resulting classification errors was generated and applied to four common classifiers (i.e., RF, SVM, LDA, KNN). It is important to note that the classification procedure using the R-package *optBiomarker* proposed by Khondoker et al. [34] was carried out. More recently, for example this approach has been used to characterize the biological pathways associated with bacterial infection [40].

**Fig 9:**
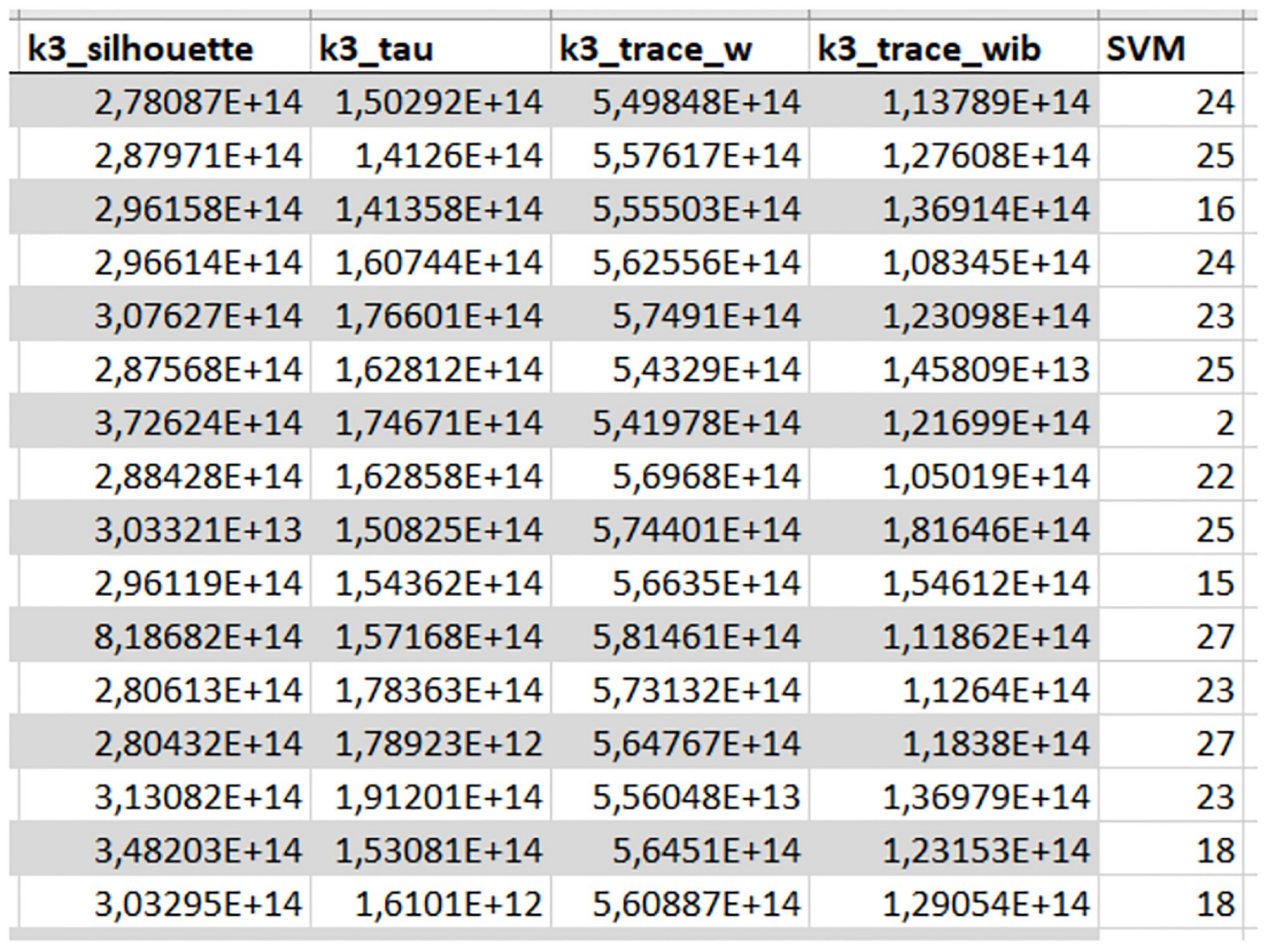
Meta dataset example. Exemplary segment of the meta dataset of simulation *S*1 including cluster indices and the SVM classification error (last column).

Fig 9 depicts an exemplary segment of the meta dataset for simulation *S*1 including cluster indices (independent variables) and the SVM classification error (dependent variable of the regression problem).

In general, the classification error and the accuracy as its counterpart are two common measures that can be applied if the class distribution is balanced (i.e., *nGr1* ≈ *nGr2*). Finally, the classification error was predicted using two popular regression models (i.e., linear and random forest regression model).

**Fig 10:**
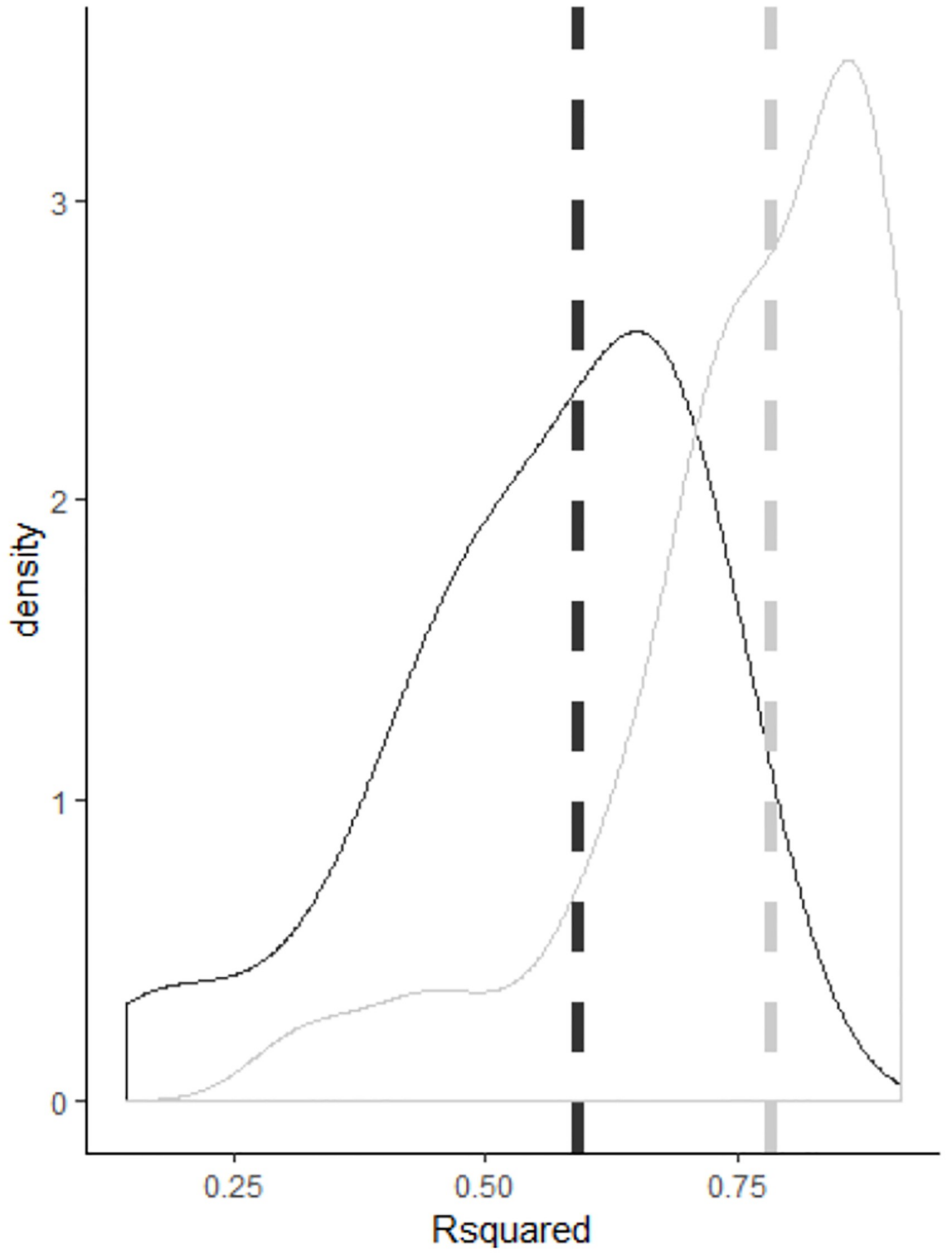
Comparing performance of regression models. Density plot of *R*^2^ values comparing linear (black) and random forest regression model (gray). The vertical lines represent the median values indicating considerable higher values for the random forest regression model.

The single steps of the workflow are based on established statistical methods. The novelty of this approach is the sophisticated combination and quantitative evaluation of the structure of the synthetic and biomedical datasets used. The *R*^2^ values of the synthetic datasets obtained with a random forest regression model (median *R*^2^ of 0.78) were significantly higher compared with the linear regression model (median *R*^2^ of 0.59, *p* < 0.01, see also Fig 10). The smallest *R*^2^ values were determined for simulation *S*9, which is characterized by higher variance and smaller effect size.

It is well known that the predictive ability in terms of classification error heavily depends on the overall discriminatory ability of the dataset. The *R*^2^ values resulting from the prediction of the classification error of the microarray dataset were smaller. This can be explained by the fact that the results of learning methods become more stable with larger datasets [41]. However, our results demonstrate that the predictability (i.e., the extent of predicting the discriminatory ability) of classification performance with respect to *R*^2^ also depends on the discriminatory ability. This means that smaller classification errors can be better estimated compared to larger errors. The high predictability of SVM errors can be explained by the stable generalization performance of the classifier due to its maximum margin property. The low predictability of KNN errors is due to its fundamental properties, since KNN does not create a learning model, also called “lazy learner”.

For the synthetic datasets, the features describing the univariate discriminatory ability, yielded the highest ranks for predicting the final classification error in our experiments. In particular, the total number of variables with p-values less than our defined thresholds (i.e., *npthTotalSum*) falls within the set of the three top ranked features for all four classifiers. The highest ranked feature for LDA classification method was the standard deviation of the t-statistics and could be explained by similar metrics based on the population mean (see also Eq 1). Analyzing the synthetic datasets, we found a high correlation between the feature rankings. The smallest coefficient of correlation was obtained in simulation *S*2 and *S*5 (see also Fig 5). In general, the correlation between *S*5 and other simulations tended to be lower. Note that *S*5 is characterized by smaller fold changes overall.

The majority of top ranked features of the microarray dataset also represent the good discriminatory ability. In particular, characteristic features of the dataset using the t-value of a Student’s t-test were identified as the most important factors for predicting the classification accuracy. Again, predicting KNN results was difficult with respect to smaller *R*^2^ values for the random forest regression model.

In summary, our results clearly demonstrate that the proposed workflow allows the prediction of the performance of classifiers before applying a learning model. Consequently, the traditional approach of comparing the performance values of different classification models can significantly be simplified and accelerated by using the proposed method. Our approach therefore has the potential to outperform the traditional approach by suggesting a particular classification algorithm that otherwise might not have been considered.

Our application examples show that the predictability strongly depends on the general predictive ability of the dataset (i.e., number and strength of discriminators). In order to predict the classification errors we recommend a random forest regression model which has a significantly better performance than the linear regression and Bayesian model.

Furthermore, the results of feature selection indicate that only a small number of features, e.g., describing the univariate discriminatory ability, are important to adequately predict the performance of classification methods, also with little computational effort which is also evident from our use case. From the viewpoint of applicability, the prediction workflow can be used manyfold, e.g. for the development of an expert system or tools for diagnostic and prognostic applications in medicine and life sciences. However, an important requirement is the availability of datasets to train and validate the model. An expansion and availability of public data repositories in biomedicine would be helpful to further improve the generalizability of such heavily data-driven approaches. In addition to these practical benefits, our workflow contributes to a better understanding of the linkage of dataset properties and the performance of a learning model needed for feature selection and classification tasks in biomarker discovery. The next logical steps of our work include analyzing the impact of hyperparameter tuning of the learning models and associated parameters, such as the size of the tuning grid, and its application to other important biological or biomedical problems to demonstrate the generalizability of this new method. Another interesting research direction raising from our results concerns the prediction of the optimal architecture of neural networks. In particular, deep neural networks have recently gained a lot of research interest in many areas [42].

## Supporting information

S1 Table Performance values in terms of *R*^2^ for prediction classification error of RF, SVM, LDA and KNN using linear regression, random forest regression and Bayesian Generalized Linear Model models for the microarray dataset. *R*^2^ values were calculated using an evaluation set.
(PDF)
