# Supplementary material for: Predicting prediction: A systematic workflow to analyze factors affecting the classification performance in genomic biomarker discovery
Source: PLoS One. 2022 Nov 9;17(11):e0276607. doi: 10.1371/journal.pone.0276607 (PMC9645616; doi:10.1371/journal.pone.0276607)
Supplement: S1 Table — R2 values were calculated using an evaluation set. (PDF) [file pone.0276607.s001.pdf]

	R^2		
**Model**	**Linear Regression**	**Random Forest**	**Bayesian GLM**
RF	0.08	0.91	0.53
SVM	0.03	0.80	0.30
LDA	0.25	0.87	0.81
KNN	0.00	0.23	0.45
